# Immune correlates of Varlilumab treated cancer patients are consistent with CD27 costimulatory activity

**DOI:** 10.1186/2051-1426-2-S3-P100

**Published:** 2014-11-06

**Authors:** Timothy Bullock, Hillary McClintic, Se Jeong, Kelly Smith, Walt Olson, Venky Ramakrishna, Laura Vitale, Jennifer Green, Michael Yellin, Thomas Davis, Tibor Keler

**Affiliations:** 1 Dept of Pathology, University of Virginia, Charlottesville, VA, USA; 2 Dept of Surgery, University of Virginia, Charlottesville, VA, USA; 3 Celldex Therapeutics, Inc., Charlottesville, VA, USA

## 

Varlilumab is a human IgG1 agonist anti-CD27 antibody designed to activate T cells through CD27 costimulation. Preclinical studies have shown that Varlilumab efficiently activates human T cells when combined with T cell receptor stimulation, enhances antigen specific CD8 T cell responses in human CD27 transgenic mice when combined with vaccination, and mediates anti-tumor activity in these mice when challenged with syngeneic tumors. A multi-dose, dose-escalation/expansion trial of Varlilumab in patients with advanced solid tumors (n = 56) or lymphoma (n = 24) has demonstrated a good safety profile with no MTD reached through the 10 mg/kg dose level. Evidence of clinical activity includes a complete response in a treatment-refractory Stage IV Hodgkin's patient (remains in remission at 12.9 months); a renal cell carcinoma (RCC) patient with a partial response (ongoing at 3.8 months); an additional RCC patient with extended stable disease (ongoing at 22.4+ months); and 12 additional patients with stable disease (range: 2.7+ to 14 months). We performed extensive correlative immune monitoring from serum and peripheral blood cells, particularly in melanoma and RCC patients. Differential gene expression analysis was assessed pre- and post-treatment by hybridizing RNA isolated from whole blood PAXgene RNA tubes onto Illumina HT-12 microarrays. Transcriptomic analysis showed significant impact of Varlilumab on immunological pathways, including T cell receptor signaling that was observed at early time points (day 2 and day 8 post treatment). Interestingly, these changes were most significant at low dose levels (0.1 and 0.3 mg/kg), however, a larger sample size would be required to confirm these results. Varlilumab administration was associated with enhanced T cell activity by evaluation of activation markers and functional analysis using IFN-γ Elispot. In particular, evaluation of response to peptides derived from melanoma antigens in selected patients showed evidence that Varlilumab promoted CD8^+ ^T cell function against melanoma antigens. Specifically, expanded responses were detected to epitopes from gp100 and MART-1, while de novo responses to epitopes MAGE-A1, NY-ESO-1 and gp100 were evident. These responses were confirmed by MHC-multimer staining. These data, and our previous analyses demonstrating a transient increase in pro-inflammatory cytokines (IP-10, IL-6, MCP-1), up-regulation of HLA-DR expression on T cells, increase in NK cell numbers, and decrease in regulatory T cells in response to Varlilumab treatment, show a pattern consistent with CD27 costimulation. These results, together with our preclinical data, provide the rationale for initiating clinical studies of Varlilumab in combination with therapies such as vaccines, checkpoint inhibitors and targeted therapies.

